# Lipid profile and dyslipidemia among school-age children in urban Ghana

**DOI:** 10.1186/s12889-018-5196-0

**Published:** 2018-03-06

**Authors:** Anna Lartey, Grace S. Marquis, Richmond Aryeetey, Helena Nti

**Affiliations:** 1 Department of Nutrition and Food Science, University of Ghana, Legon, Ghana; 2 School of Human Nutrition, McGill University, 21,111 Lakeshore Road, Ste-Anne-de-Bellevue, QC H9X 3V9 Canada; 3 School of Public Health, University of Ghana, Box LG 13, Legon, Ghana

**Keywords:** Lipid profile, Dyslipidemia, School, Child, Cholesterol, Ghana

## Abstract

**Background:**

Dyslipidemia during childhood has been associated with higher risk of atherosclerosis later in life. Information on the lipid profile of Ghanaian children is scarce. The aim of this study was to assess the lipid profiles of school children between the ages of 9–15 years, living in urban Ghana.

**Methods:**

A total of 802 randomly selected school-age children participated in the Ghana School Survey implemented in Kumasi and Accra, Ghana. A structured questionnaire was used to collect information on child and maternal socio-demographic characteristics (including age, education, and occupation), 7-day food frequency, home and school activity, as well as measurement of weight and standing height. Weight, height, and age data were converted into BMI-for-age indices to determine weight status. Finger-prick fasting blood samples were taken from the school-age children. Total cholesterol (TC), triglyceride (TG), high density lipoprotein (HDL-C) and low-density lipoprotein (LDL-C) cholesterol levels were determined using the CardioChek® PA Test System. Reference lipid levels based on the US National Cholesterol Education Program 2001 guidelines were used to determine the proportion of children with dyslipidemia.

**Results:**

The mean TC, LDL-C, HDL-C, and TG levels were 149.0 ± 57.0 mg/dl, 80.1 ± 38.6 mg/dl, 53.5 ± 19.4 mg/dl, and 71.4 ± 54.7 mg/dl, respectively. Mean TC/HDL-C ratio was 3.0 ± 1.0. The proportion of children with abnormal values were 12.1% for TC, 4.5% for TG, 28.4% for HDL-C, 9.2% for LDL-C, and 6.6% for TC/HDL-C ratio. The levels of dyslipidemia (HDL, LDL, and TC/HDL-C ratio) were higher among overweight/obese compared to normal-weight children. More frequent fruit consumption was also linked with lower LDL-C (*p* = 0.020) while watching television (TV) in the mornings was linked with both higher TC (*p* = 0.011) and TG (*p* = 0.006).

**Conclusions:**

Majority of urban-dwelling Ghanaian school children had normal lipid profiles. However, the higher levels of dyslipidemia observed among overweight and obese children suggest the need for population level physical activity and dietary interventions among children to reduce risk of cardiovascular diseases in adult life.

## Background

Cardiovascular diseases (CVD), formerly believed to be common only among affluent societies are now becoming leading causes of death in developing countries such as Ghana. The Ghana Health Service estimates that CVDs were the leading cause of institutional deaths in 2008, accounting for 14.5% of all reported deaths [1]. A study at the Korle-Bu Teaching Hospital indicated that CVDs constituted more than one-fifth of all causes of death from 2006 to 2010 [2]. Dyslipidemia, particularly in terms of cholesterol and triglyceride levels, has been identified as an important risk factor of CVD. Abnormalities in lipoprotein metabolism represent about 50% of the population-attributable risk of developing CVDs [3]. Consequently, both cardiovascular risk assessment and CVD management are based on the blood levels of these lipids, particularly, low-density lipoprotein cholesterol (LDL-C) [4–6].

Available evidence indicates that atherosclerosis, the process that subsequently leads to CVD, starts in childhood and progresses gradually into adulthood. The Bogalusa Heart Study and the Pathobiological Determinants of Atherosclerosis in Youth (PDAY) Research, both conducted in the United States of America, showed that high concentrations of low density lipoprotein cholesterol (LDL-C) and low levels of high density lipoprotein cholesterol (HDL-C) in children and youth were associated with higher risk of atherosclerosis later in life [7, 8]. As such, it is important that efforts to reduce CVD should start during childhood.

Little is known about the lipid profile of children and adolescents in Ghana. One study that was conducted in the Ga-East Municipality showed a low level of hypercholesterolemia (2.8%) among overweight and obese school children [9]. However, the study did not provide data on the levels of LDL-C and HDL-C as well as the ratio of total cholesterol (TC) to HDL-C, which is considered as a more sensitive and specific index of cardiovascular risk [10]. Evidence of dyslipidemia among children is needed to inform the development of strategies as well as resources needed to address CVDs. The objective of this study was, therefore, to determine the prevalence of dyslipidemia among school-going children in urban Ghana. Evidence from the study will be useful for understanding blood lipid situation among school children in Ghana, as a first step to designing appropriate public health response.

## Methods

### Study participants

This study was part of the Ghana School Survey that was designed to assess the prevalence and determinants of overweight and obesity among school-aged children in urban Ghana (Aryeetey et al. 2017 [11]). The study population included 802 school children between the ages of 9–15 years who were recruited between December 2009 and February 2012 from 121 schools located in Accra and Kumasi, the two largest cities in Ghana. To obtain the sub-sample that was used in the present study, each overweight or obese child who was recruited was matched with a child of normal Body Mass Index (BMI) of the same age and sex. To be eligible to participate in the study, children must be between the ages of 9–15 years. The study was approved by the Ethical Review Boards of McGill University, Canada (A09-B21-09A) and Noguchi Memorial Institute for Medical Research, University of Ghana, Legon (004/09–10). Permissions were obtained from the Ghana Education Service and Heads of all participating schools before data collection. Written informed consent was obtained from all participating children and their parents at recruitment into the study. Only children who expressed their willingness to participate and obtained signed parental consent were included.

### Data collection

A wide variety of data were collected in the study as reported elsewhere [11]. Data included in the current analysis were socio-demographic characteristics of the school children as well as caregivers/parents dietary behavior, physical activity, anthropometric measurements and biomarkers of blood lipids. Briefly, a structured questionnaire was used to collect information on caregiver and child age as well as caregiver education, occupation, and household living situation, including ownership of a list of home assets. A food-frequency questionnaire was used to describe the 7-day frequency of consumption of a list of at least 60 foods and beverages. Physical activity was assessed with structured questions on participation in sports, household chores and routine transportation to and from school. Body weight and height of children were measured at the respective school premises. Participants were required to remove all heavy clothing and accessories such as shoes or sandals, belts, watch, and sweaters, and their pockets were emptied prior to being measured. Body weight was measured to the nearest 0.1 kg using the Tanita Digital Scale (model BWB-800, Tanita Corporation, USA). Height measurements were taken to the nearest 0.1 cm using the Shorr Board (Shorr Productions, Olney, MD). All measurements were done and recorded in duplicate. Weight and height measurements were converted to body mass index-for-age Z-scores (BMIZ) using the World Health Organization’s AnthroPlus 2009. Overweight/obesity was defined as BMIZ > 1.0 [12].

For children identified for the lipid profile assessment, parents were informed the day before sample collection that their children had been selected for the lipid profile determination. They were requested to ensure that children did not eat any food before coming to school the following day. They were also to ensure that the child ate supper latest by 7 pm the day before the blood draw. On the day of the lipid profile determination, the children were to report to school early, by 8 am. After the tests had been completed, the children were provided with breakfast by the project (comprising of bread, and 200 ml of malted cocoa beverage).

On the day of the lipid determination, each participating child was asked whether he/she had eaten breakfast or any food or drink that morning. If the child had eaten breakfast that morning, they were not included and therefore no finger prick blood was taken. Finger prick blood samples were collected only on children who had not eaten any breakfast that morning and were in 8–12 h overnight fast. The procedure for the finger prick blood collection was explained to the child and asked if they consented to the procedure. It the child indicated they did not want to continue, he or she was excluded. Upon consent, the tip of the index finger was first cleaned with alcohol-soaked cotton ball before taking the finger-prick blood. A drop of the whole blood was placed on the lipid strip and inserted in the CardioChek® PA Test System (Indianapolis, U.S.A) following the manufacturer’s guidelines. The lipid profile including total triglycerides (TG), total cholesterol (TC), high density lipoprotein cholesterol (HDL-C), and low density lipoprotein cholesterol (LDL-C) were determined using the CardioChek® PA Test System (Indianapolis, U.S.A).

Trained interviewers administered the questionnaire to the school children to collect socio-demographic information about the child and his or her household. All data collection was carried out between December 2009 and February 2012.

### Data analyses

Descriptive statistics of continuous variables were presented as means and standard deviation and categorical variables as percentages. The percentage of children with abnormal lipid concentrations was calculated using the reference values described in the US National Cholesterol Education Program 2001 Guidelines [6]. The following values were classified as abnormal lipid concentrations: TC ≥ 200 mg/dl, LDL-C ≥ 130 mg/dl, HDL-C ≤ 40 mg/dl, and TG ≥ 150 mg/dl. The ratio of TC to HDL was calculated for each participant and children with values greater than 4.5 considered to be at borderline or high cardiovascular risk [10, 13]. The Student’s t-test for Independent samples was used to compare the mean lipid concentrations of overweight/obese and normal-weight children. Multilevel mixed-effects linear regression models were fitted to generate unbiased effect estimates and standard error for covariates of blood lipid indicators. All statistical analyses were conducted using SAS (version 9.2, Cary, NC, USA) and Stata (Version 12, StatCorp, TX, USA) and statistical significance was considered at *p* <  0.05.

## Results

Data for 802 children aged 9–15 years were included in the current analyses. Of these, more than 60% were females (Table 1). The mean age of the participating children was 12.2 ± 1.6 years. Generally, there were no differences in demographic characteristics of normal-weight and overweight/obese children; educational level of the mothers of overweight/obese children was higher compared to normal-weight children. Dietary and physical activity behaviors were similar across overweight children and those who are no (Table 2). The only exceptions concerning dietary and activity were frequency of breakfast skipping (*p* = 0.013), motorized transportation to school (*p* = 0.002) and participation in sports (*p* = 0.001).

**Table 1:** Socio-demographic characteristics of school age children and their caregivers/mothers

	Normal-weight (*n* = 409)	Percent	Overweight/obese^1^ (*n* = 393)	Percent	*P*-value^2^
Child Characteristics					
Age (years)	12.2 ± 1.6		12.1 ± 1.6		0.15
BMI-for-age z score	−0.1 ± 0.6		1.9 ± 0.7		< 0.01
Female sex n(%)	257	62.8	265	67.4	0.143
Attending public school, n(%)	140	58.6	99	41.4	0.057
Maternal/Caregiver Characteristics^3^					
Age (completed years)	40.7 ± 9.1		41.3 ± 9.3		0.49
Education (years)	9.6 ± 4.3		10.5 ± 4.4		0.01
Occupation^4^ (%)					017
Professional	39	13.1	58	18.9	
Office worker	8	2.7	10	3.3	
Artisan	38	12.7	44	14.4	
Trading	196	65.8	172	56.2	
Unemployed	17	5.7	22	7.2	
Socioeconomic status^5^ (%)					0.17
Low	121	41.4	100	33.9	
Medium	133	45.6	153	51.9	
High	38	13.1	42	14.2	

Values are presented as mean ± SD or n (%)

^1^Defined as BMI-for-age Z-score > 1

^2^Based on Student's t-test for means or Chi-Square test for proportions

^3^Total of 198 missing data (normal-weight = 111, overweight/obese = 87)

^4^Professionals included teachers, lawyers, doctors, and accountants, etc. Office workers included secretaries and office clerks

^5^SES score was determined based on summed scores of household income, ownership of vehicle, mother and father’s educational level. Scores were classified as follows: 0–1 = low, 2–4 = medium, and 5–6 = high socioeconomic status

**Table 2:** Dietary and physical activity habits of Ghanaian children 9–15 years

	Normal-weight (*n* = 409)		Overweight/obese^1^ (*n* = 393)		*p*-value
	n	%	n	%	
Dietary habits					
Skip breakfast	100	23.3	112	29.9	0.069
Access to soft drinks at home	133	31.0	121	32.4	0.681
Breakfast < 3 days/week	37	8.6	53	14.2	0.013
Sweetened drink ≥3 times/week	370	86.2	317	84.4	0.550
Fruit consumption (3 times/week)	132	30.8	118	31.6	0.811
Vegetable consumption (3 times/week)	166	38.7	146	39.0	0.921
Physical activity					
Transport to school ≥3 days/week	210	49.0	224	59.9	0.002
Household chores > 5 times/week	279	65.0	246	65.8	0.826
Any sporting activity ≥3 times/week	138	32.2	82	21.9	0.001
Sedentary behavior					
Watch television ≥5 times/week	293	68.3	242	64.9	0.305
Watch television in morning before school	48	11.2	39	10.4	0.729

^1^Defined as BMI-for-age Z-score > 1

Except for HDL-C, fasting blood lipid concentrations of most of the school children were within the normal ranges of reference values (Table 3). The mean TC, LDL-C, HDL-C, and TG concentrations were 149.0 ± 57.0 mg/dl, 80.1 ± 38.6 mg/dl, 53.5 ± 19.4 mg/dl, and 71.4 ± 54.7 mg/dl, respectively. There were no differences between boys and girls in terms of fasting blood lipid concentrations, except the level of TC among the overweight/obese children (Table 3). Overweight/obese children had significantly higher levels of TG (76.6 ± 60.8 mg/dl v 66.5 ± 47.8 mg/dl, *p* = 0.009) and LDL-C (85.1 ± 41.7 mg/dl v 75.4 ± 34.9 mg/dl, *p* = 0.004), and lower HDL-C levels (51.3 ± 19.8 mg/dl v 55.7 ± 18.9 mg/dl, *p* = 0.0011) compared to normal-weight children. Additionally, overweight/obese children tended to have greater mean TC concentration than those with normal weight, although the difference was not statistically significant (152.8 ± 62.6 mg/dl versus 145.3 ± 50.9 mg/dl, *p* = 0.0627). Although the mean TC to HDL-C ratio indicated that the school age children were at low cardiovascular risk, overweight/obese children had a significantly higher ratio (3.2 ± 1.0 v 2.8 ± 0.9, *p* <  0.001) compared to normal weight children.

**Table 3:** Blood lipid levels of school-going urban Ghanaian children aged 9–15 years, classified by weight status

Blood lipid indicators	Normal-weight (*n* = 409)	Overweight/obese^1^ (*n* = 393)	*p*-value^2^
	Mean ± SD	Mean ± SD	
Total cholesterol (mg/dl)			
Boys	140.3 ± 56.6	160.4 ± 72.1	0.063
Girls	148.3 ± 47.1	149.2 ± 57.2	
Total	145.3 ± 50.9	152.8 ± 62.6	
LDL^3^ cholesterol (mg/dl)			
Boys	72.9 ± 39.8	91.7 ± 49.5^a^	< 0.001
Girls	76.9 ± 31.6	81.8 ± 36.9	
Total	75.4 ± 34.9	85.1 ± 41.7	
HDL^4^ cholesterol (mg/dl)			
Boys	53.9 ± 19.6	52.2 ± 21.7	0.001
Girls	56.8 ± 18.4	50.8 ± 18.8	
Total	55.7 ± 18.9	51.3 ± 19.8	
Total cholesterol/HDL cholesterol ratio			
Boys	2.8 ± 0.9	3.3 ± 1.0	< 0.001
Girls	2.8 ± 0.8	3.1 ± 1.0	
Total	2.8 ± 0.9	3.2 ± 1.0	
Triglycerides (mg/dl)			
Boys	67.9 ± 43.8	82.3 ± 58.3	0.009
Girls	65.7 ± 50.1	73.9 ± 61.8	
Total	66.5 ± 47.8	76.6 ± 60.8	

^1^Defined as BMI-for-age Z-score > 1

^2^Comparison of blood lipids across normal-weight and overweight/obese children using Student’s t-Test for Independent Samples

^3^Low-density lipoprotein, ^4^High-density lipoprotein

^a^The difference between boys and girls in the same group was significant (*p*-value = 0.0453)

More than one-quarter of the children (28.4%) had HDL-C levels that were indicative of high or borderline cardiovascular risk (Table 4). Additionally, hypercholesterolemia (indicated by elevated total cholesterol) was indicated in more than 10%. The levels of dyslipidemia were significantly different between overweight/obese and normal-weight children (Table 4), but not between boys and girls.

**Table 4:** prevalence of dyslipidemia among school-going urban Ghanaian children aged 9–15 years, classified by weight status

Type of dyslipidemia^a, b^	Normal-weight (*n* = 409)		Overweight/obese (*n* = 393)		*p*-value
	N	%	n	%	
High total cholesterol					
Boys	17	11.2	20	15.6	0.067
Girls	24	9.3	36	13.4	
High LDL cholesterol					
Boys	12	7.9	16	12.5	0.042
Girls	17	6.7	28	10.7	
Low HDL cholesterol					
Boys	38	25.0	46	35.9	< 0.001
Girls	55	21.4	89	33.6	
High TC/HDL cholesterol ratio					
Boys	4	2.6	14	10.9	0.002
Girls	12	4.7	23	8.7	
High triglycerides					
Boys	7	4.6	10	7.8	0.030
Girls	5	1.9	12	5.3	

^a^Based on US National Cholesterol Education Program 2001 Guidelines: Total cholesterol > 200 mg/dl, Low-density lipoprotein cholesterol > 130 mg/dl, High-density lipoprotein cholesterol ≤40 mg/dl, triglycerides > 150 mg/dl

^b^TC/HDL-C ratio cut-off value for borderline to high risk, Kocaoglu et al. [13]: Total cholesterol/HDL-C ratio ≥ 4.5

In multivariate regression modelling, TC was linked with watching television in the morning prior to going to school (*p* = 0.011) (Table 5). Overweight status predicted a lower HDL-C concentration (*p* = 0.001). On the other hand, overweight predicted a higher LDL-C (*p* = 0.002). LDL-C was also linked with child age (*p* = 0.010), less frequent consumption of fruit (*p* = 0.020) and watching television in the morning (*p* = 0.006).

**Table 5:** Risk factors linked with blood lipid indicators among school age children in Ghana

Explanatory variables	coefficient	*p*-value	95% Confidence Interval	
			Lower bound	Upper bound
Total Cholesterol				
Fixed effects				
Sex (female)	−3.388	0.513	−13.531	6.755
Age, yr	2.015	0.218	−1.189	5.219
Obese^1^	7.808	0.108	− 1.716	17.333
School type^2^	−8.024	0.311	−23.535	7.486
Watch television in the morning	12.114	0.011	2.8319	21.397
Random effects^3^				
Region	26.621		9.608	73.757
Schools	23.678		17.378	32.262
High Density Lipoprotein				
Fixed effects				
Sex (female)	−1.547	0.317	−4.576	1.483
Age, yr	−.502	0.298	−1.447	0.443
Obese	−4.632	0.001	−7.480	−1.785
School type	−0.330	0.872	−4.336	3.675
Random effects				
Region	11.913		4.415	32.147
Schools	5.058		3.372	7.588
Triglycerides				
Fixed Effects				
Sex(female)	−7.135	0.215	−18.410	4.140
Age	−0.997	0.583	−2.566	4.560
Obese	1.763	0.744	−8.814	12.340
School type	−7.728	0.382	−25.062	9.605
Systolic blood pressure	0.568	0.013	0.118	1.018
Watch television in the morning	14.464	0.006	4.145	24.782
Random effects				
Region	6.589		1.109	39.158
Schools	26.594		18.402	38.432
Low Density lipoprotein				
Fixed effects				
Sex (female)	4.870	0.321	−4.745	14.486
Age	3.937	0.010	0.950	6.923
Obese	14.486	0.002	5.195	23.777
School type	3.348	0.625	−10.083	16.778
Eating fruit 3 or more times in a week	−10.583	0.020	−19.508	−1.659
Watch television in the morning	14.464	0.006	4.145	24.782
Random effects				
Region	7.757		2.046	29.411
Schools	17.184		12.007	24.592
Triglyceride-High Density Lipoprotein Ratio				
Fixed effects				
Sex (female)	0.070	0.453	−0.113	0.253
Age	0.070	0.012	0.015	0.125
Obese	0.445	< 0.001	0.281	0.609
School type	0.107	0.454	−.173	0.386
Random effects				
Region	0.149		0.036	0.614
School	0.437		0.309	0.617

Other variables controlled in model: maternal education, maternal and paternal occupation, systolic blood pressure, child fruit and vegetable consumption, activity (including means of transportation to school, engagement in sporting activity, performing household chores), and sedentary behavior (watching television in mornings)

^1^BMIZ = Body Mass Index-for-Age Z-Score

^2^Categorized as public or private school

^3^Represents variation in region and school

## Discussion

This study provides information on the lipid profile of school-going children in urban Ghana. With the exception of HLD-C, the levels of lipids and lipoproteins of the children were mostly within the desirable values. In a majority of school children (71.6%), the concentration of HDL-C was below the level that presents a negative risk factor for CVD (≥ 60 mg/dl) [5]. The observed mean HDL-C concentration was lower than what was reported among 13–19 year old children in Tunisia (58.0 mg/dl), but higher when compared to the HDL-C levels of school children of similar age range in Turkey (49.0–49.8 mg/dl) and Brazil (41.1–48.7 mg/dl) [14–17]. These geographical differences could be due to various factors including genetics, diet, and physical activity habits of the children. Related to the low HDL-C levels is the observation that the most prevalent type of dyslipidemia was in relation to HDL-C, a finding that is similar to studies conducted in Sri Lanka (23.3%) and Brazil (29.5%) [16, 18]. However, in a study among younger Brazilian children (5–8 years) lower prevalence of impaired HDL-C concentration (17.2%) was reported [19].

The observed low HDL-C levels in our study suggest that school-going children in urban Ghana may be at marginal risk of CVD later in life. In Spain and Japan, high levels HDL-C in children was given as a potential explanation for the relatively low Coronary Heart Disease mortality rates compared to other developed countries [20, 21]. Evidence from large epidemiological studies suggests that each 1 mg/dl increase in HDL-C is associated with a decrease of 2–3% in the risk of coronary artery disease [22]. The protective effect of HDL-C may be due to its role in reverse cholesterol transport and its ability to prevent the oxidation of LDL-C [23].

The level of lipids and lipoproteins in both children and adults can be affected by a number of factors, including diet and physical activity which are modifiable behaviors. Although dyslipidemia can result from genetic disorders such as homozygous familial hypercholesterolemia, it usually results from secondary type which is often related to sub-optimal lifestyles [24]. A study in Brazil that included overweight and obese children reported a positive association between elevated TC (β = 0.36, *p* = 0.04) and the consumption of full fat dairy products, as well as between elevated TG (β = 0.017, *p* = 0.04) and the percentage of total energy obtained from saturated fat [17]. Studies also indicate that while high intakes of fats and proteins as percentages of total energy increase TC and LDL-C levels, a higher percentage of carbohydrates than the recommended macronutrient range is associated with low HDL-C levels [25, 26]. In Greece, HDL-C level was inversely associated with a lifestyle of higher consumption of sugar-sweetened beverages, more screen time (e.g. television watching), and shorter sleep duration among 9–13 year old children [27]. Additionally, having more eating events and higher levels of physical activity was inversely associated with TC (β = − 0.064, *p* = 0.006), LDL-C (β = − 0.065, *p* = 0.004), and TC to HDL-C ratio (β = − 0.049, *p* = 0.049) in the multivariate models. Physically active lifestyle has been shown to improve HDL/LDL-C ratio through an increase in lipoprotein lipase activity [28].

The intake of sugar-sweetened drinks among Ghanaian school children is high, with an average consumption of about 314 ml per day recorded among 8–18 year old school children [9]. Additionally, majority of the children (60.9%) in that study engaged in less than 60 min of physical activity daily. Less than one-third of the school children who participated in the Global School-based Student Health Survey engaged in sporting activities at least three times in a week [29]. There is, therefore, need for interventions that will improve lifestyles, particularly dietary and physical activity habits of children and adolescents as part of comprehensive strategy to reducing the risk of CVD later in life.

Our study did not find significant differences in the lipid profiles of boys and girls, with the exception of TC among overweight/obese children. The available literature on the sex differences in the lipid profile of school children provides inconsistent results [30]. Among 5–14 year old Colombian children, a greater proportion of girls had undesirable levels of TC (7.9% v 3.0%, *p* < 0.05), LDL-C (11.6% v 4.7%, *p* < 0.05), and TG (6.9% v 5.7%, *p* < 0.05) compared to their male counterparts [31]. Similar finding was reported by Ghannem et al. among 13–19 year old Tunisian children [15]. On the other hand, one study in Brazil did not observe any differences in the lipid levels of boys and girls [16], similar to our findings. Another study in Brazil reported a sex difference in HDL-C levels only, TC, LDL-C, and TG levels were similar for boys and girls [19].

In the present study, overweight and obese children had significantly undesirable levels of lipids compared to normal weight children. This finding is consistent with reports from other settings. In Tunisia, the BMI of 13–19 year old children correlated with TC (*r* = 0.13, *p* < 0.0001) [15]. Manios et al. reported that among Turkish school children, overweight boys had significantly higher level of TC, LDL-C, TC/HDL-C ratio, and TG, and overweight girls had lower HDL-C levels compared to their normal weight counterparts [32]. Similar results have been reported among school children in Greece, Spain, and the United States of America [33–35]. These findings indicate the clustering of CVD risk factors among children. Considering that modifiable factors such as diet and physical activity levels are associated with both obesity and lipid profile, lifestyle changing interventions that begin in childhood and adolescence can be helpful in reducing the cardiovascular risks later in life.

A strength of this study is that it provides comprehensive lipid profile of Ghanaian school children who live in urban settings. However, it is limited in that we focused on a narrow set of CVD risk factors. Thus, the study does not provide data on other previously identified risk factors such as fasting glucose, insulin resistance, blood pressure, and smoking habits of the children [36–38].

## Conclusions

In conclusion, majority of school children living in urban Ghana had desirable lipid profiles. There was, however, a substantial proportion with low levels of HDL-C. Additionally, higher levels of dyslipidemia were observed among overweight/obese children. Possible explanations for this observation are the high consumption of sugar-sweetened beverages and low level of physical activity that have been reported among the study population by earlier studies. Thus, interventions that aim to improve the diet and physical activity levels during childhood and adolescence may be needed to reduce the cardiovascular risk later in life.

## Abbreviations

BMI: Body mass index
BMIZ: body mass index-for-age Z-scores
CVD: Cardiovascular diseases
HDL-C: High density lipoprotein cholesterol
LDL-C: Low-density lipoprotein cholesterol
PDAY: Pathobiological determinants of atherosclerosis in youth
TC: Total cholesterol
TG: Triglyceride
TV: Television
